# Long-Term Patterns of Online Evidence Retrieval Use in General Practice: A 12-Month Study

**DOI:** 10.2196/jmir.974

**Published:** 2008-03-19

**Authors:** Farah Magrabi, Johanna I Westbrook, Michael R Kidd, Richard O Day, Enrico Coiera

**Affiliations:** ^5^Clinical PharmacologyTherapeutics CentreSt Vincent’s HospitalSydneyAustralia; ^4^Physiology & PharmacologySchool of Medical SciencesFaculty of MedicineUniversity New South WalesSydneyAustralia; ^3^Discipline of General PracticeUniversity of SydneySydneyAustralia; ^2^Health Informatics Research & Evaluation UnitFaculty of Health SciencesUniversity of SydneySydneyAustralia; ^1^Centre for Health InformaticsUniversity of New South WalesSydneyAustralia

**Keywords:** Clinical informatics, information retrieval, evidence-based medicine, family practice, evaluation studies, Internet

## Abstract

**Background:**

Provision of online evidence at the point of care is one strategy that could provide clinicians with easy access to up-to-date evidence in clinical settings in order to support evidence-based decision making.

**Objective:**

The aim was to determine long-term use of an online evidence system in routine clinical practice.

**Methods:**

This was a prospective cohort study. 59 clinicians who had a computer with Internet access in their consulting room participated in a 12-month trial of Quick Clinical, an online evidence system specifically designed around the needs of general practitioners (GPs). Patterns of use were determined by examination of computer logs and survey analysis.

**Results:**

On average, 9.9 searches were conducted by each GP in the first 2 months of the study. After this, usage dropped to 4.4 searches per GP in the third month and then levelled off to between 0.4 and 2.6 searches per GP per month. The majority of searches (79.2%, 2013/2543) were conducted during practice hours (between 9 am and 5 pm) and on weekdays (90.7%, 2315/2543). The most frequent searches related to diagnosis (33.6%, 821/2291) and treatment (34.5%, 844/2291).

**Conclusion:**

GPs will use an online evidence retrieval system in routine practice; however, usage rates drop significantly after initial introduction of the system. Long-term studies are required to determine the extent to which GPs will integrate the use of such technologies into their everyday clinical practice and how this will affect the satisfaction and health outcomes of their patients.

## Introduction

Good quality online evidence retrieval systems should provide clinicians with convenient access to up-to-date, reliable, and pertinent information at the point of care. While the potential of online evidence systems in providing information to answer clinical questions has been demonstrated in controlled laboratory settings [1], their impact on clinicians’ decision-making behavior is dependent on uptake and sustained use in an everyday clinical setting. Of the few investigations of online evidence use in routine clinical work, the majority have measured usage by clinicians a few weeks following provision of the system [2,3]. There are few long-term assessments beyond the initial period of introduction, during which the perceived novelty of the intervention is likely to affect patterns of use.

We sought to measure the long-term use of an online evidence retrieval system, Quick Clinical (QC), in routine general practice settings. In a previous 4-week study conducted from October to November 2002, 193 general practitioners (GPs) used the QC online evidence system to perform an average of 8.7 searches per month [4]. The majority of these searches (81.1%) were conducted from consulting rooms during office hours. The most frequent searches related to diagnosis (37.3%) and treatment (32.1%). Search topics included a broad spectrum of diseases, including common conditions such as asthma, diabetes, and hypertension. In this paper we present the results of a 12-month trial of QC in general practice.

## Methods

### Setting and Participants

A total of 59 GPs from across Australia participated in the trial. Clinicians who had a computer with Internet access in their consulting room were recruited via a call for volunteers advertised in journals, newsletters, and a clinician listserv.

### Quick Clinical

**Figure 1:**
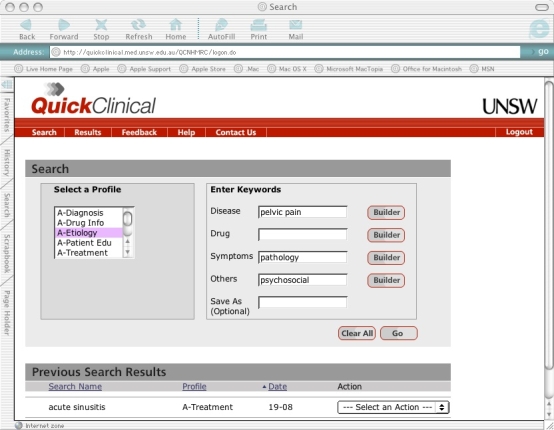
User interface of QC

QC is based on the generic use of search filters explicitly designed to meet the information needs of specific user groups. The filters can be customized to meet the varying needs of different groups [5]. Search filters were adjusted for this study to provide five “profiles” specifically designed for GPs: disease etiology, diagnosis, treatment, prescribing, and patient education. Users first select a search filter or profile that matches their question type (eg, diagnosis, treatment) and then enter keywords that more specifically describe their query. Up to four types of keywords can be used in association with a given profile: disease, drug, symptoms, other. For example, a clinician who encounters a 32-year-old woman with a fourth presentation of pelvic pain in the last 6 months but whose physical examination, ultrasound studies, and swabs for infection are all negative, may have a question regarding the social, psychological, as well as biological causes of pelvic pain. The clinician could select the “etiology” profile and enter “pelvic pain,” “pathology,” and “psychosocial” as keywords (Figure 1). The search filters retrieve evidence from information resources selected for local relevance, including PubMed, MIMS (a pharmaceutical database), Therapeutic Guidelines, Merck Manual, and HealthInsite (a government-funded health database for consumers [6]). Users can also search each of these resources individually.

### Procedures

Clinicians were asked to use QC in their practice from May 2005 to April 2006. QC was available via a standard Web browser interface (eg, Firefox, Microsoft Internet Explorer). Each participant obtained a personal username and password to access QC and completed an online tutorial on how to use the system. A help manual was also available online. All participants were asked to complete an online survey about their computer use during consultations, and demographic information was also sought at the beginning of the study. We did not send out any reminders or prompt participants to keep using QC. Frequency and purpose of system use were determined from automatically generated computer logs used to record details of each search, including the search filter chosen, keywords entered, data sources accessed, and the date, time, and duration of the searches.

The study was recognized by the Royal Australian College of General Practitioners (RACGP) for its continuing medical education (CME) program. Education points were not directly linked to the number of searches performed, but to trial completion. Ethics approval for the protocol was received from the ethics committees of the University of New South Wales, University of Sydney, and RACGP.

### Analysis

Statistical analysis of data from the computer logs and online survey was undertaken using SPSS (Statistical Package for the Social Sciences) v11.0 (SPSS Inc, Chicago, IL, USA). Descriptive statistics were used to examine patterns of use and responses to the online survey. Comparisons between groups were made using Student *t* test and chi-square analyses.

## Results

### Participants

A total of 140 GPs expressed interest in using QC in their practice. Of these, only 59 (42%) completed the online registration and tutorial enabling them to use the system. The majority of participants were male (71%, 42/59), aged 35-54 years, and 71% (42/59) obtained their primary medical qualification in Australia. Most GPs worked in a group practice or medical center, and 56% (33/59) were fellows of the RACGP (Table 1). The majority (83%, 42/59) worked in an accredited practice, and 78% (46/59) had 11 or more years experience in primary care. On average, participants worked 34.16 hours per week in direct patient care (SD = 10.48) and consulted with 4.07 patients per hour (SD = 0.69).

**Table 1:** Demographics of study GPs in comparison to the general population of Australian GPs in 2005/2006

Characteristic	%	
	Study GPs (N = 59)	Australian GPs^*^ (N = 953)
**Gender** (*χ*^2^_1_ = 0.3, *P* = .60)		
Male	71	67.9
Female	29	32.1
**Age** (*χ*^2^_3_ = 3.8, *P* = .28)		
< 35 years	8	8.9
35-44 years	32	25.5
45-54 years	37	31.8
55+ years	22	33.8
**Country of graduation** (*χ*^2^_1_ = 0.04, *P* = .83)		
Australia	71	69.9
Overseas	29	30.1
**Fellow of RACGP** (*χ*^2^_1_ = 4.2, *P* = .04)	56	42.3
**Practice type** (*χ*^2^_1_ = 1.2, *P* = .28)		
Group or medical center	83	87.8
Solo	17	12.2
**Computer use during consultations** (*χ*^2^_4_ = 1.3, *P* = .87)^†^		
Prescribing	100	89.5
Medical records	93	76.4
Internet	80	72.9
Other administrative purposes (eg, appointments)	78	79.8
Email	75	72.9
Patient education	95	62.9^‡^
Online evidence	81	16.6^‡^

^*^Data are for Australian GPs in 2005/2006 [7].

^†^N = 880 for computer use data among Australian GPs [7].

^‡^N = 1061 for patient education and online evidence data among Australian GPs [8].

All participants reported having a computer on their desk where they saw patients. All but one used their computer during consultations, and 88% (52/59) indicated having “good” to “excellent” computer skills. Computers were used for a range of practice functions, including prescribing, medical records, practice administration, Internet, email, patient education, and online evidence. Of those who knew their Internet connection type, 89% (47/53) reported having access via a broadband connection.

### Patterns of QC Use

**Figure 2:**
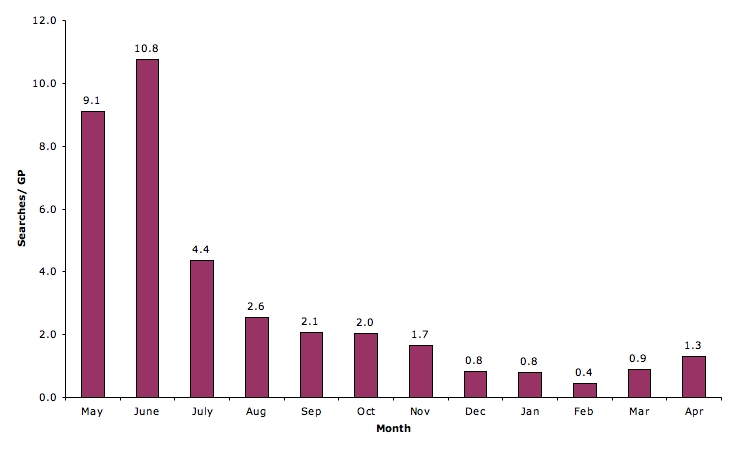
Average number of monthly QC searches over 12-month study period (N = 2543 searches)

**Figure 3:**
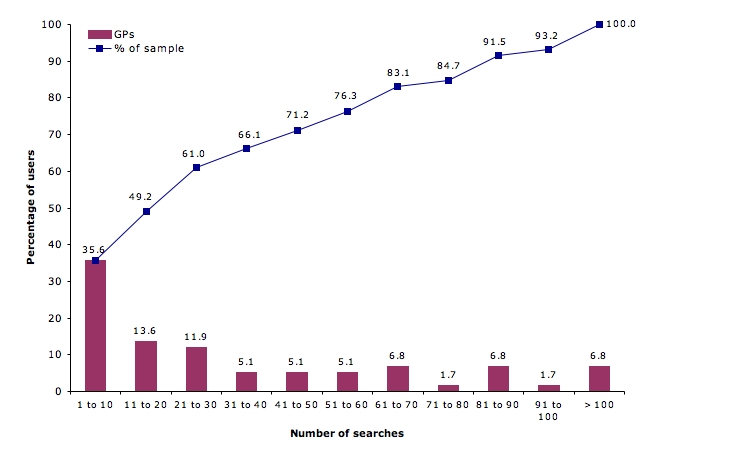
Percentage of GPs conducting QC searches over the 12-month study period, by number of searches (N = 59 GPs)

In total, participants conducted 2543 searches over the 12-month period (May 2005 to April 2006). The total number of searches conducted by each participant ranged from 1 to 240 over the trial (mean_59_ = 39.14, SD = 45.29; median_59_ = 23); 9 participants did not use QC after the first 2 months of the study (mean_50_ = 38.28, SD = 38.80; median_50_ = 28). Relatively higher rates of use were recorded in the initial 2 months of the study (Figure 2). On average, 9.1 to 10.8 searches were conducted by each GP during this period. After this, the usage rate dropped to 4.4 searches per GP in the third month and then levelled off to between 0.4 and 2.6 searches per GP per month. There was significant variation in individual use of the system (Figure 3). We compared the group of participants who used QC for less than 10 searches (36%, 21/59), the “low” use group, with those who used the resource 50 or more times (29%, 17/59), the “high” use group. There was no difference in the makeup of the high and low use groups by gender, years of general practice experience, place of graduation, practice type, RACGP fellowship status, or information-seeking behavior (Table 2). However, the low use group had a significant number of participants aged 45 years and older (*χ* ^2^_1_= 4.8, *P* = .03).

**Table 2:** Comparison of QC high and low use groups

Characteristic	% (No.)	
	Low Use^*^ (N = 21)	High Use^†^ (N = 17)
**Gender** (*χ*^2^_1_ = 0.6, *P* = .44)		
Female	24 (5)	35 (6)
Male	76 (16)	65 (11)
**Age** (*χ*^2^_1_ = 4.8, *P* = .03)		
< 45 years	19 (4)	53 (9)
45+ years	81 (17)	47 (8)
**Country of graduation** (*χ*^2^_1_ = 0.2, *P* = .64)		
Australia	57 (12)	65 (11)
Overseas	43 (9)	35 (6)
**Experience in general practice** (*χ*^2^_1_ = 1.4, *P* = .24)		
≤ 10 years	10 (2)	24 (4)
11+ years	90 (19)	76 (13)
**Practice type** (*χ*^2^_1_ = 0.2, *P* = .70)		
Group or medical center	76 (16)	71 (12)
Solo	24 (5)	29 (5)
**Fellow of RACGP** (*χ*^2^_1_ = 0.1, *P* = .80)		
Yes	43 (9)	47 (8)
No	57 (12)	53 (9)
**Search for information during consultations** (*χ*^2^_1_ = 0.4, *P* = .54)		
Yes	81 (17)	88 (15)
No	19 (4)	12 (2)

^*^Low use is 1-10 searches.

^†^High use is ≥ 50 searches.

**Figure 4:**
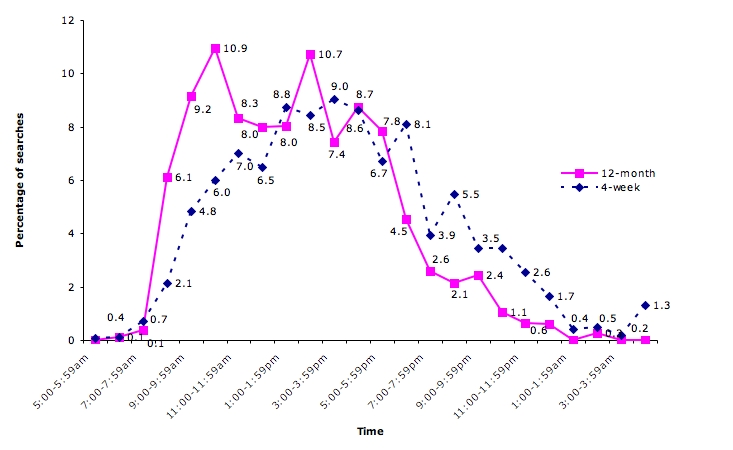
QC use by time of day (12-month N = 2543; 4-week N = 1257 searches)

**Figure 5:**
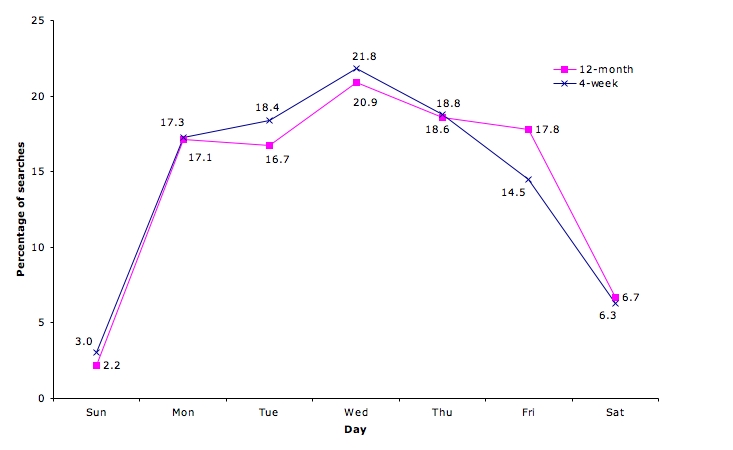
QC use by day of the week (12-month N = 2543; 4-week N = 1293 searches)

QC use varied throughout the day. The system was mostly used during practice hours, peaking in the morning and afternoon sessions; 79% (2013/2543) of the searches were conducted between 9 am and 5 pm (Figure 4). The use of the system also varied over the work week, peaking on Wednesday; 91% (2315/2543) of the searches were conducted between Monday and Friday (Figure 5). Thus, some use also occurred outside work hours.

### Search Patterns

A large proportion of searches (90%, 2291/2543) were undertaken using a QC profile. Of these, 33.6% (821/2291) related to questions about diagnosis, 34.5% (844/2291) to treatment, and 13.8% (337/2291) to patient education. Almost 5% (117/2291) were related to prescribing and 6.0% (147/2291) to disease etiology. Disease-specific keywords were used to describe clinical questions in a significant proportion of the searches (72.9%, 1854/2543). In comparison, few searches utilized keywords related to drugs (6.5%, 165/2543) or symptoms (8.8%, 224/2543). The fourth keyword type, “other,” was utilized in 19.3% (491/2543) of the searches. The 10 most frequently used entries for each keyword category are listed in Table 3.

**Table 3:** Top 10 keywords used to describe clinical questions (N = 2543 searches)

Disease (n = 1854)	Drug (n = 165)	Symptoms (n = 224)	Other (n = 491)
asthma	self-examination	pain	prevention
maculopathy	Implanon	abdominal	cholecystectomy
dermatitis	vitamins	itch	breast self-examination
ovarian cancer	folic acid	nocturia	pregnancy
gout	isotretinoin	upper ab	females
cholecystectomy	mirtazapine	lumps	diet
acne	flucloxacillin	pain	inheritance
cholecystitis	prednisolone	gallstone	surgery
sudden infant death syndrome	dietary supplement	hematur	dietary sources
breast cancer	saw palmetto	lump	child

## Discussion

### Main Findings and Implications

This is the first study to directly measure individual GPs’ long-term patterns of use of an online evidence facility. We found that QC was used mostly during weekday practice hours. On average, each clinician conducted 0.7 searches per month over the trial. The majority of the searches related to questions about diagnosis and treatment. These findings indicate that the QC model fits into general practice and that GPs will use online evidence past a typical 1- or 2-month trial.

On average, each clinician used QC for one search every 2 months. Although the use of electronic resources is reported to be growing steadily, a recent review confirms that GPs still prefer to consult their colleagues and textbooks over electronic resources to answer their clinical questions [9]. In a study of clinicians’ actual information-seeking behavior, Ely et al [10] found online sources to be the third most frequently used resource after textbooks and humans. Depending on practice variables and methods used to measure the frequency of clinical questions, it is estimated that GPs generate up to two questions per consultation [9]. Given that clinicians typically pursue only a small proportion of their clinical questions, the current long-term frequencies of online evidence use are likely to be low.

After an initial surge in the use of QC during the first 2 months of the study, utilization by each GP levelled to 0.6 searches per month on average. This could be attributed to the natural tendency for usage rates to drop as the novelty of the system wears off. Lack of easy access may also account for low rates of use as participants were required to log on to QC each time they used it. The use of other search systems may have been a confounding variable. At the beginning of the study, 81% (48/59) of participants reported that they used online evidence during consultations, indicating that online information seeking was not a novel practice to them and that QC may have been used alongside a range of other online resources they regularly consulted. As this was not a controlled study, other variables might have affected use independent of QC.

A significant proportion of the low use group was 45 years or older (Table 2); it is possible that this group may have been less comfortable with online searching despite their reports of having good to excellent computer skills. It is also likely that changes in practice conditions may have impacted QC use. Only 59 of the 140 GPs who initially registered completed the study. Relocations and changes in practice conditions may have impacted participation in this study. However, in a follow-up of a previous study of QC in which we specifically examined factors associated with integration of online evidence into clinical practice, we found that levels of use could only be directly linked to clinicians’ experiences of improvement in patient care as a result of using QC [11].

There are few studies of the type and quality of online resources used by clinicians. In a laboratory study that allowed participants to choose their own electronic resources to answer simulated clinical questions, investigators found that despite the availability of high-quality resources such as Clinical Evidence and Cochrane, Google and other Internet sites were used at the same rate as Medline (22.6%) and accounted for the second most frequently used resources after UpToDate (65.9%), a resource presenting concise summaries of clinical evidence [12]. Increasing use of general purpose search engines to retrieve online information of variable quality is likely to impact the quality and safety of clinical decisions, a trend worthy of monitoring.

### Comparison With Existing Literature

Little comparative data are available as there are few studies of online evidence retrieval use in general practice. Clinicians’ use of QC beyond the initial 2 months is comparable to studies of Medline use in hospital and ambulatory settings. Thus, eight out of 10 studies reviewed by Hersh [2] reported utilization rates ranging from 0.3 to 3.5 searches per person-month over 2 to 36 months. Our study data are comparable to these data. The two outliers are both short-term studies: Collen and Flagle’s 2.7-month study reported 6.7 searches per person-month [13], and Osheroff and Bankowitz’s 2-week study reported 12.5 searches per person-month [14]. In contrast with clinicians’ self-reports of online information seeking, where 45% of searching was reported to occur outside practice hours [15], we found that QC was largely used during practice hours (79% of searches were conducted between 9 am and 5 pm).

While use of QC went down after 4 weeks, the overall pattern remained similar in terms of days and times when searches were undertaken. General patterns of QC use observed in the current study are consistent with a 2002 trial of the system over 4 weeks [4]. We found no difference in the overall utilization pattern by time of day (see Figure 4) or day of the week (*χ* ^2^_6_= 9.7, *P* = .14, see Figure 5). However, there was a significant difference in QC profile use; while the proportion of diagnosis and etiology questions was similar, a larger proportion of questions in the 12-month study related to diagnosis and patient education, and fewer related to prescribing (14.9%, *χ* ^2^_4_= 35.7, *P* < .001). As in the previous 4-week trial, there was considerable variation in use of QC among individual clinicians. While short-term trials are adequate for predicting broad patterns of online evidence use, long-term studies are still necessary to measure the overall uptake and integration into clinical practice.

### Limitations of This Study

The participants were a self-selected cohort who volunteered to participate in the study. In the pre-trial survey, eight out of 10 GPs within this group (81%, 48/59) reported using online evidence during consultations and may therefore have been predisposed to using QC in their practice compared to the general population (17%, see Table 1). The majority of participants were new to QC; however, some (29%, 17/59) reported using the system in the previous 2002 study. When compared to the general population of Australian GPs, there was no difference in gender, age, or place of graduation. Participants’ overall computer use was found to be representative of that in the general population of GPs within Australia. Though education points were linked to trial completion and not the number of searches performed, recognition of this study as a CME activity is likely to have resulted in a significantly higher proportion of RACGP fellows within our sample. On the whole, the demographics of our cohort were generally comparable to the general population of Australian GPs.

### Conclusion

This study measured GPs’ individual use of an online evidence retrieval system over a 12-month period. Clinicians used the system in routine care to answer questions mostly about diagnosis and treatment. Usage rates dropped significantly after initial introduction of the system. While short-term trials are adequate for measuring broad patterns of online evidence use, overall uptake and integration into clinical practice require long-term studies.

## Abbreviations

CME: continuing medical education
GP: general practitioner
QC: Quick Clinical
RACGP: Royal Australian College of General Practitioners
